# Fabrication of patterned graphitized carbon wires using low voltage near-field electrospinning, pyrolysis, electrodeposition, and chemical vapor deposition

**DOI:** 10.1038/s41378-019-0117-7

**Published:** 2020-01-13

**Authors:** Derosh George, Adrian Garcia, Quang Pham, Mario Ramos Perez, Jufeng Deng, Michelle Trang Nguyen, Tuo Zhou, Sergio O. Martinez-Chapa, Yoonjin Won, Chong Liu, Roger C. Lo, Regina Ragan, Marc Madou

**Affiliations:** 1 Mechanical and Aerospace Engineering, University of California, Irvine, USA; 2 Chemical Engineering and Materials Science, University of California, Irvine, USA; 3 Materials and Manufacturing Technology, University of California, Irvine, USA; 4 School of Engineering and Sciences, Tecnologico de Monterrey, Monterrey, Mexico; 5 Mechanical Engineering, Dalian University of Technology, Dalian, China; 6 Chemical Engineering, California State University, Long Beach, USA; 7 Present Address: Mechanical Engineering, Centro de Enseñanza Técnica y Superior Universidad, Mexicali, Mexico

**Keywords:** Nanowires, Nanowires

## Abstract

We herein report a high-resolution nanopatterning method using low voltage electromechanical spinning with a rotating collector to obtain aligned graphitized micro and nanowires for carbon nanomanufacturing. A small wire diameter and a small inter-wire spacing were obtained by controlling the electric field, the spinneret-to-collector distance, the pyrolysis parameters, the linear speed of the spinneret, the rotational speed of the collector. Using a simple scaling analysis, we show how the straightness and the diameter of the wires can be controlled by the electric field and the distance of the spinneret to the collector. A small inter-wire spacing, as predicted by a simple model, was achieved by simultaneously controlling the linear speed of the spinneret and the rotational speed of the collector. Rapid drying of the polymer nanowires enabled the facile fabrication of suspended wires over various structures. Patterned polyacrylonitrile wires were carbonized using standard stabilization and pyrolysis to obtain carbon nanowires. Suspended carbon nanowires with a diameter of <50 nm were obtained. We also established a method for making patterned, highly graphitized structures by using the aforementioned carbon wire structures as a template for chemical vapor deposition of graphite. This patterning technique offers high throughput for nano writing, which outperforms other existing nanopatterning techniques, making it a potential candidate for large-scale carbon nanomanufacturing.

## Graphitized carbon: spinning wires

Electromechanical spinning enables the fabrication of highly graphitized carbon wires with diameters down to tens of nanometers. Graphitized carbon has a number of attractive properties for functional devices, and in wire-form can be used for strain, gas and temperature sensing, as well as for far-field thermal emitters. However, current fabrication processes for graphitized carbon wires are limited by their low yields and complexity. Now, a team led by Marc Madou from the University of California, Irvine reports a high-throughput near-field electrospinning process for creating polymer wire patterns that can then be converted to pyrolytic carbon, followed by chemical vapor deposition to obtain highly-graphitized carbon. Controlling the electrospinning process allows wire diameters to span from tens of nanometers up to microns.

## Abstract (web summary)

The schematic representation of the fabrication process and SEM micrographs of carbon nanowires.

## Graphical abstract

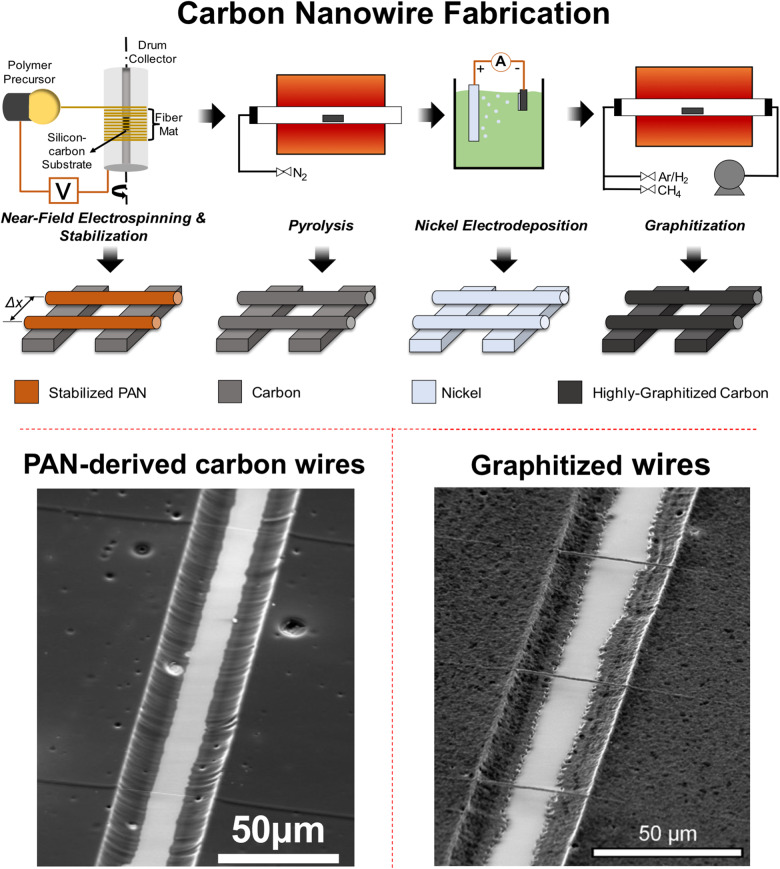

## Introduction

Graphitized carbon has become a preferred material for numerous applications because of its superior physicochemical properties, such as mechanical strength^1,2^, electrochemical activity^3–5^, and exceptional thermal and electrical conductivities^6^. More specifically, carbon-based micro/nanopatterns are important for developing electrochemical sensors^7^, transparent conductors^8^, flexible electronics^9^, photonic crystals^10^, and supercapacitors^11^. Techniques including patterning photoresist followed by pyrolysis (C-MEMS and C-NEMS), ozone-assisted etching, and self assembly are used to fabricate patterned carbon structures^7,11,12^. However, the pyrolysis of a polymer carbon precursor, commonly used technique for C-MEMS and C-NEMS, results in non-graphitizable carbon^13^. Despite its innate non-graphitic properties, a recent study has revealed that the glassy carbon derived from some of the carbon precursors can be transformed into “glassy graphene” by using the material itself as a template^14^. Alternatively, more graphitic carbon can be obtained by controlling the precursor manufacturing process. The latter graphitization method is based on mechanical stressing and stabilization of the polymer precursor before pyrolysis^15^. Yet, chemical vapor deposition (CVD) of carbon on a patterned nickel template remains one of the most popular ways to fabricate patterned graphitized structures because of the better understood chemistry of the growth process^9,16,17^. However, this patterning technique has not reached its full potential due to the lack of a scalable and affordable micro/nanopatterning technique to achieve patterned nickel structures.

Apart from their potential use as a carbon source to synthesize patterned graphitized carbon structures, carbon patterns are involved in a myriad of applications, including microneedles, stem cell scaffolds, and gas sensing platforms^18–20^. Patterned precursor polymers shrink isometrically to the final carbon structures after pyrolysis. Taking advantage of this isometric transformation, carbon structures of various shapes are made by patterning photopolymers using any type of lithography followed by pyrolysis. Other methods, including micro-transfer molding and micro/nano writing, have been reported for patterning polymer precursors for making carbon structures^21,22^. Among the various explored shapes, producing three-dimensional structures, e.g., suspended structures, remains a challenging task. Although far field electrospinning has been used to deposit suspended wires onto a patterned surface, the morphology of the deposited wires is restricted by the design of the collector^23^. Recent studies have pursued the rational creation of complex multi-dimensional carbon shapes in order to further expand the applications of structurally designed carbon-based materials^22^. Sub-micron carbon structures have been made by pyrolyzing polymer structures created by two-photon lithography. Various other works using photolithography-based fabrication methods have also been employed to make controlled patterns of wires with submicron dimensions^24,25^. However, a technique for controlled high-throughput fabrication of carbon wires in the nanometer regime (<100 nm) with intended morphology is yet to be developed and is required to exploit the full potential of the carbon nanostructures^26^.

Among various shapes, micro- and nano-wires have gained unprecedented attention from the research community owing to their potential applications in various fields, such as photonics^27^, electronics^28^, energy harvesting^29^, and metamaterials^22^. It has been reported that the fabrication of wires having structural uniformity could lead to low-loss photonic wires for waveguiding^30^. Through a layer-by-layer assembly of nanowires, fabrication of multi-nanowire field effect transistors (FETs) can be realized^31^. Assembly of inorganic nanowires may be used for the fabrication of flexible electronics^32^. In another reported case, it has been shown that the conversion of mechanical energy to electrical energy is possible via an array of zinc oxide nanowires^33^. Mechanical metamaterials made of polymers can be transformed into carbon to enhance its mechanical properties^22^. Apart from these general applications of nanowires, aligned and suspended carbon wires have found applications, such as strain sensors, temperature sensors, and gas sensors^26^, and are expected to have potential applications in far-field thermal emitters^34^. Nonetheless, their potential use in next-generation products remains limited by the low throughput, high complexity, small working area, and high costs of the equipment involved in current fabrication techniques.

Far-field and near-field electrospinning, on the other hand, have the potential to become scalable nanomanufacturing methods for nanowire structures owing to their simplicity, affordability, and versatility. While far-field electrospinning (FFES) utilizes the regime where bending instabilities generate randomly oriented nanowire mats, near-field electrospinning (NFES) exploits the regime where the bending instabilities are minimal, and thus precise patterning applications are allowed^35^. To further improve the controllability of NFES, our team developed a low voltage electromechanical spinning (EMS) method^36,37^, which allows for even more controlled nano writing with various polymers in NFES^38,39^. However, there still remains a strong need for a high-resolution, high-throughput patterning method, applicable to a wider variety of polymer precursors, to manufacture thinner wires for C-MEMS and C-NEMS. In addition, an improved throughput of the NFES will help in manufacturing new classes of well-defined polymer and carbon micro/nanostructures for advanced applications in electrochemical sensing, energy storage, and stem cell research^18,40–45^.

Herein, we report on a high-resolution patterning method using EMS with a rotating collector, followed by a combination of scalable pyrolysis and electrodeposition to obtain either aligned glassy carbon or graphitized sub-micron carbon wires for carbon nanomanufacturing. Wire diameters as small as 37.15 ± 1.5 nm and inter-wire spacings as small as 7.9 ± 2.3 μm were manufactured by controlling the electric field, spinneret-to-collector distance, linear speed of the spinneret, rotational speed of the collector, and pyrolysis parameters. A nickel catalyst layer for producing highly graphitized carbon was produced by the galvanostatic electrodeposition of a thin conformal coating around the carbon wires. After CVD on the metalized carbon wires in a reducing environment, we generated patterned carbon structures consisting of highly graphitized carbon.

## Results

### Scaling analysis

Wire patterning using NFES involves two main steps, i.e., jet initiation and controlled wire deposition. In the presence of an electric field *E*, jet initiation occurs at the surface of a liquid droplet with diameter *a* and surface tension *γ*, when the electrostatic pressure $(\sim \!\!{\it{\epsilon }}_oE^2)$ is comparable to the capillary pressure $(\sim \!\!\frac{\gamma }{a})$, where ${\it{\epsilon }}_o$ is the permittivity of free space. Therefore, in order to overcome the surface tension of the liquid droplet to form a Taylor cone on the droplet, an electric field $E\sim \frac{V}{L}\sim \sqrt {\frac{\gamma }{{{\it{\epsilon }}_oa}}}$(~10^6^ V/m, considering typical values *γ* ~ 0.1 Nm, a ~ 0.01 m and ${\it{\epsilon }}_o$ = 8.85 × 10^−12^ F/m) needs to be applied, where *L* is the distance between the droplet and the collector, and *V* is the voltage applied across *L*. At a low voltage, wires can be extruded out of the droplet by bringing it close to the collector, while for a case with a relatively large *L*, a higher voltage is needed to be applied to initiate the electrospinning. Once a continuous wire jet is obtained, the relative motion of the spinneret and the collector is used to control the deposition of wires.

NFES is carried out in the straight jet regime, where electrically driven bending instabilities caused by the lateral perturbations are absent, to provide better control over the nanowire deposition. In this regime, where electrically driven instabilities are absent, mechanical instabilities such as buckling could still be present. The deposition process in this regime is analogous to lowering an elastic rope onto a surface, a process that is accompanied by buckling and subsequently coiling of the rope^46^. To avoid this buckling and thus to obtain straight wires, the distance *L* has to be small and the collector velocity must be more than or equal to the jet velocity. For a liquid of conductivity K and density $\rho$, the jet velocity, *v*_jet_, is reported to be $\left( {\frac{{\pi {\rm{K}}\gamma }}{{{\it{\epsilon }}_o\rho }}} \right)^{\frac{1}{3}}\frac{1}{{f^2}}$, where *f* is the nondimensionalized radius of the jet^47^. From this expression for the jet velocity, it is observed that smaller wires are generated with higher velocity due to its inverse square relationship with the wire radius. This high velocity poses a significant challenge, when trying to obtain straight and controlled writing of smaller wires, which requires the collector to be mounted onto an expensive high-precision high-speed linear stage. We overcame this hurdle by translating this demanding linear motion into a rotational motion *ω* and by stepping the position of the wire jet to obtain aligned nanowires on a collector of radius *R*.

The rotational motion is mainly used here to uncoil the wires, not to thin the wires. This is evident from the order of the force per unit length $(\sim \!\!\rho r^2\omega ^2R)$ exerted on the extruded nanowires by the rotating collector. For instance, when a collector rotates at 600 RPM (*ω* = 10 Hz), a typical value used in our experiments, the force acting on the nanowires is relatively small (~10^−10^ N/m) compared to the electrostatic force per unit length $(\sim \!\! \sigma Er)$ generated with an applied electric field of $10^5{\mathrm{V}}/{\mathrm{m}}(\sim \!\!10^{ - 8} \, {\mathrm{N}}/{\mathrm{m}})$^48^. As far as the opposing forces are concerned, the inertial force per unit length $(\sim\!\! \rho r^2\frac{{v_{\mathrm{jet}}^2}}{L})$ is more significant than the combination of the surface tension and viscous force^49^. Due to the dominant inertial forces acting on the wire, the wire radius can be estimated by equating the electrostatic force to the inertial force, as given in Eq. (1);

1 $$\frac{{\rho v_{\mathrm{jet}}^2r}}{L}\sim \sigma E$$

Assuming the flow of current (*I*) is mainly driven by the motion of the extruded charged wire, the surface charge density σ scales as $\sigma \sim \frac{I}{{v_{\mathrm{jet}}r}}$. Thus, Eq. (1) is approximated as $\frac{{\rho v_{\mathrm{jet}}^2r}}{L}\sim \frac{{IE}}{{v_{\mathrm{jet}}r}}$. The value of the current *I* is reported to be of the order of $100Ev_{\mathrm{jet}}^{\frac{1}{2}}rK^{\frac{2}{5}}$ nA^50^. So, the radius of the wire scales as:

2 $$r\sim \frac{{100V^2{\rm{K}}^{\frac{2}{5}}}}{{\rho v_{\mathrm{jet}}^{\frac{5}{2}}L}}\mathrm{nm}$$

Ultimately, the wire diameter is controlled either by changing the applied voltage (*V*) or the distance between spinneret and collector (*L*). Unlike previously reported FFES studies, it is expected here that a lower voltage results in smaller wires^51,52^. The formation of smaller nanowires and finer control of wire deposition at lower voltage make rotational NFES a superior approach for nanopatterning. Additionally, this rotational motion can improve the inter-wire spacing for high-resolution nanopatterning. In this set-up, the resolution ($\Delta x$) of the nanopatterning is a function of both linear velocity of the spinneret (*v*) and the rotational speed of the collector (*ω*) as given in Eq. (3):

3 $$\Delta x = \frac{v}{\omega }$$

### Parametric study of polymer wire fabrication and patterning

**Fig. 1:**
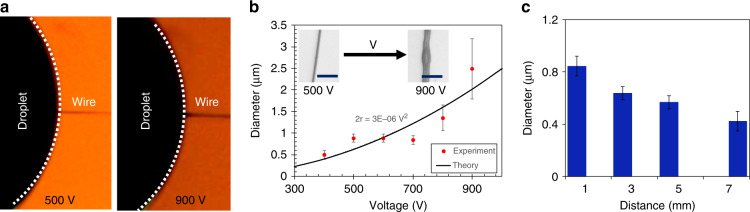
Effect of operating parameters on wire diameters. **a** Micrograph of wires at 500 and 900 V, **b** Variation of the diameter of the wire with the applied voltage (The scale bar is 7 µm long), and **c** Variation of the diameter with the distance between the spinneret and the rotating collector (L).

A polyacrylonitrile (PAN) solution of 11 wt.% was used to carry out the experiments. Wires were initiated by applying a voltage between a polymer droplet and a rotating collector and then bringing the droplet sufficiently close to the collector. Once a steady Taylor cone was established, the distance between the spinneret and the rotating collector was adjusted to the desired working distance. The effect of voltage on the diameter of the wires was investigated by fabricating wires at voltages ranging from 400 to 900 V. This range chosen here for PAN falls within other reported near field electrospinning voltages for polymers (0.2 to 12 kV)^53^. As shown in Fig. 1a, b, the experimental results demonstrate good agreement with the predicted trend that the diameter increases with applied voltage (Eq. (2)). The wire diameter showed an inverse dependency on the working distance between the spinneret and the collector, again as predicted by Eq. (2) (Fig. 1c). In this near field electrospinning setup, the larger wires were obtained by applying higher voltages, but they often came with variations in diameter along the length of the wire (inset in Fig. 1b). This inconsistency in the wire diameter was due to an insufficient rate of solvent evaporation from these wires during the transit from the spinneret to the collector.

**Fig. 2:**
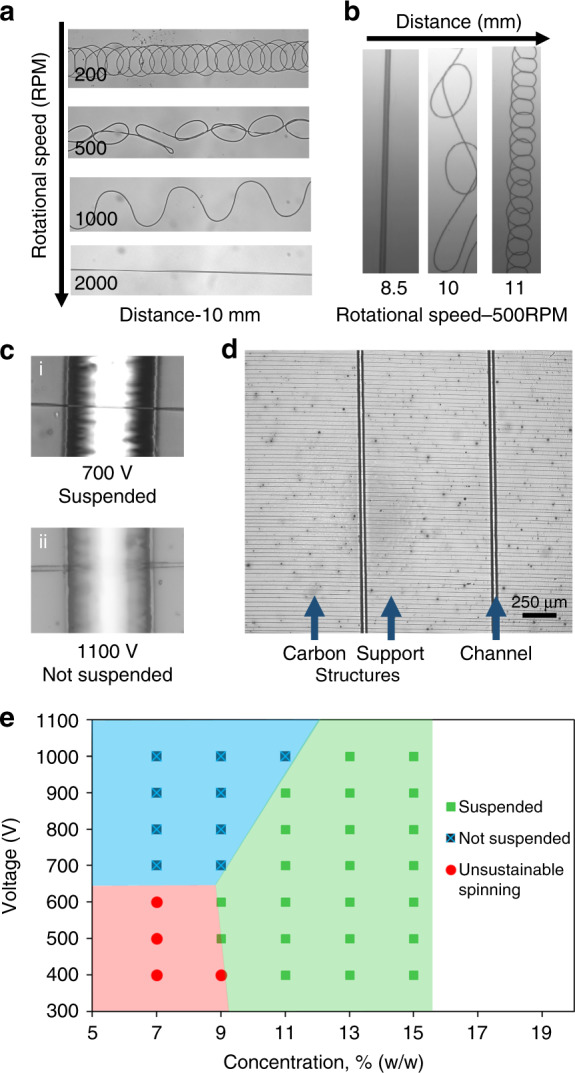
Parametric study of wire morphology. **a** Micrographs of wire profiles obtained as a function of rotational speed. **b** Micrographs of wire profiles obtained as a function of working distance (voltage = 1500 V). **c** i. Successfully formed suspended wires at low voltage. ii. No suspended wire formation at high voltage. **d** Wire patterns (horizontal lines) formed over carbon support structures with two microchannels (vertical lines). **e** The combinations of voltages and polymer concentrations which produced suspended wires (green) and non-suspended wires (blue). The red region indicates the set of parameters that cannot have a sustainable jet.

A time scale evaluation of the jet travel time and the evaporation rate gives one an insight into how important it is to work in the low voltage regime to obtain uniform wires. The jet travel time to obtain straight wires scales as $\sim \! L/R\omega \sim \left( {10^{ - 3}} \right)/\left( {10^{ - 1}} \right)\left( {10} \right) = 10^{ - 3}$ s, whereas the evaporation time scales as $\sim \!\!r^2/D$ which is approximately equal to 10^−1^s for the relatively bigger wires obtained at higher voltages. Therefore, in order to obtain solid and uniform wires, either the wires must be small (diameter ~ 100 nm) or the distance between the spinneret and collector should be far enough for the solvent to fully evaporate. However, electrospinning at a distance of about 20 mm (voltage = 1500 V) resulted in the formation of coiled wires (Fig. 2) with the current NFES setup when PAN is used. The formation of these coiled wires is attributed to the higher velocity of the wires relative to the collector^54^, which results in a higher probability of deviation from a straight path^55^. As depicted in Fig. 2a, b, a shorter spinneret-to-collector distance and/or a faster collector rotational speed must be used to achieve straight patterns. Therefore, a shorter working distance with low voltage electrospinning is suggested to obtain a patterned deposition of small wires with a consistent diameter on a flat substrate. These observations on the straightness of the wires and their uniformity of diameter confirm that the low-voltage NFES with a rotating collector is ideal for forming straight PAN nanowires for nanomanufacturing. Additionally, low-voltage electrospinning allowed for the deposition of suspended wires across support structures due to the increased strength resulting from the better solvent evaporation and thus solidification for suspended structures (Fig. 2c i). However, at relatively high voltages, the wires fell into the trenches (Fig. 2c ii). Therefore, the enhanced diffusion-based solvent evaporation rate of the wires formed using NFES enabled the patterning of suspended wires over channels as shown in Fig. 2d. With an appropriate combination of voltage (500 V) and concentration (11%), polymer wires suspended across a large gap (>5 mm) can be achieved (Fig. S2).

Fabrication of suspended wires with high concentration solutions must be easier because the formed structure can sustain its shape due to the large polymer chain density in the solution. To further verify the prediction, experiments with different concentrations of polymer solutions were performed (7, 9, 11, 13, and 15%). Concentrated solutions helped in the successful construction of suspended wires across channels as predicted. Here, the polymer solution with a low concentration of PAN had difficulty forming wires (Fig. 2e). Electrospinnability of a solution depends on inter-chain interactions of the polymer chains in the solution. Solutions with low concentration do not have enough interactions among chains and hence lack electrospinnability. It was observed that at high concentrations (>13% w/w), suspended wires formed even at high voltages (>900 V) as opposed to a lower concentration solution (Fig. 2e). Nonetheless, a high concentration solution was not considered for the fabrication of carbon nanowires since it resulted in wires of relatively larger diameter.

**Fig. 3:**
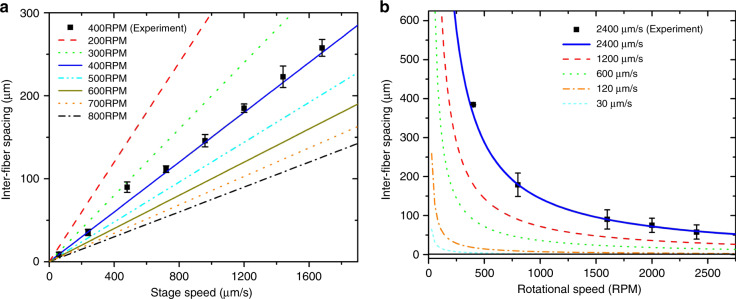
Characterization of writing resolution. Variation of inter-wire spacing with **a** stage speed and **b** rotational speed of the collector.

**Fig. 4:**
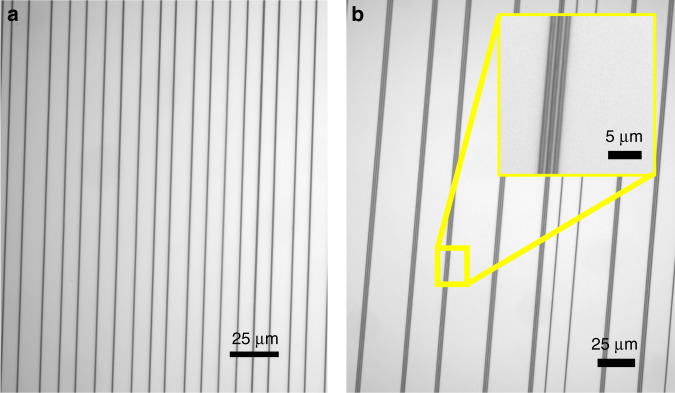
Micrographs of wire patterns at various resolutions. **a** Wires (∼400 nm) written in parallel with ∼8 µm spacing written at a speed of about 400 mm/s using NFES. **b** Non-uniform inter-wire spacing at a lower velocity (*v*). Inter-wire spacing less than 1µm was attained, albeit not uniform (Inset).

As shown in Fig. 3a, the inter-wire spacing increases linearly with the linear speed of the spinneret, which agrees well with the predictions from Eq. (3). Moreover, the resolution of the patterns (*Δx*) consistently improved with increasing rotational speed (Fig. 3b) until *Δx* reached 7.9 ± 2.3 μm (Fig. 4a). Our attempts to produce patterns with a resolution of <7 μm resulted in uneven inter-wire spacing with the smallest *Δx* < 1 μm (Fig. 4b). The irregularity of spacing distribution may be attributed to either the rotational imbalance of the collector or the air disturbance caused by the collector rotation.

**Fig. 5:**
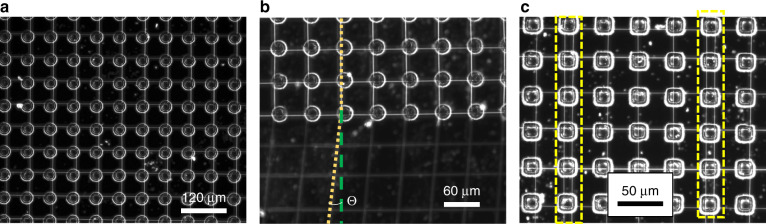
Suspended wires fabricated on pillar arrays. **a** Patterned deposition of wires on a carbon pillar array. **b** Straightening of the wires that are coming at an angle to the array. **c** Multi-wire deposition of wires on a single column when the pillar spacing is more than the expected wire spacing.

Improved alignment and better patterning can be attained by actively directing the incoming wires to desired locations. Previous studies have shown that a concentrated electric field produced by sharp locations on the substrate can be used to guide the wires^56^. We used patterned carbon pillars on a silicon substrate to collect the wires. The results showed that the pillars attracted the wires towards them to form highly ordered suspended wires on them. Experiments were performed with pillars of cylindrical and square cross-sections. A preferential deposition on the edge of the pillars was observed (Fig. 5 and S3). The observed preferential deposition was corroborated by the electric field simulations carried out with COMSOL Multiphysics, which indicated a concentrated electric field near the edges of the pillars (Fig. S2). Additionally, prior research on the pyrolysis shrinkage by Martinez et al. shows that the structures may form sharp edges after pyrolysis^57^. Those sharp edges could have further enhanced a favored deposition on the edges due to the concentrated electric field in that region. The required spacing between the wires can be obtained using Eq. (3), which should equate to the distance between the pillars. The combination of a pillar array and NFES produces suspended wires across all the pillars with predetermined inter-wire spacing (Fig. 5a). It is interesting to note that the attraction of the pillars was dominant enough to ensure that the wires were laid down exactly on the pillars even if the incoming wires were slightly offset by a small angle to the pillar array, correcting the error on its own, as shown in Fig. 5b. An inter-wire spacing lower than the pillar spacing resulted in the pairing of wires in a regular interval (Fig. 5c).

### Carbonization of nanowires

Carbon nanowires were made by stabilizing patterned PAN wires obtained using NFES, followed by pyrolysis. The stabilization step was performed at 270 ^o^C to ensure a high yield during pyrolysis^53^. In the case of suspended nanowires, heating the wires resulted in the relaxation of the wires, which caused wire sagging. The polymer wires soften when the temperature reaches its glass transition temperature^58^. When the polymer is at its glass transition temperature, the polymer chain mobility increases, and as a result, the wires relax. This polymer chain mobility and the reduction of the stress built-up on the wire from the drying process during electrospinning may have led to the wires’ sagging during stabilization. Even though suspending wires across a wide channel was possible during the electrospinning process, this sagging has made wires touch the floor of the channel. A statistical study was performed with a channel of varying widths to evaluate the effect of channel width on the ability to form suspended carbon nanowires. The studies showed an 87% survival rate of suspended wires on 7 μm-deep channels having a width < 40 μm (Table S3).

In the pyrolysis step, the polymer wires were converted to sub-50 nm carbon wires due to the shrinkage. An isometric shrinkage is expected for a free-standing polymer structure when subjected to pyrolysis. Here the wires had different mechanical constraints depending on whether it was suspended or on a planar surface, which could lead to different extents of shrinkage-/anisotropic shrinkage of the wires. A detailed study was performed to quantify the shrinkage of the wires that were suspended and on a surface. Wires were pyrolyzed at 600 ^o^C, 700 ^o^C, 800 ^o^C, 900 ^o^C, and 1000 ^o^C, respectively, to investigate the effect of the temperature on the shrinkage. Results showed an increasing trend in the percentage of shrinkage from 47.7% for the wires pyrolyzed at 600^o^C to 74.6% for those on the surface at 1000 ^o^C (Fig. S4). This shrinkage was small as compared to the suspended part of the wires. The percentage of shrinkage for the suspended region ranged from 58.2% to 88.6% for a pyrolysis temperature ranging from 600 ^o^C to 1000 ^o^C, respectively (Fig. S3).

**Fig. 6:**
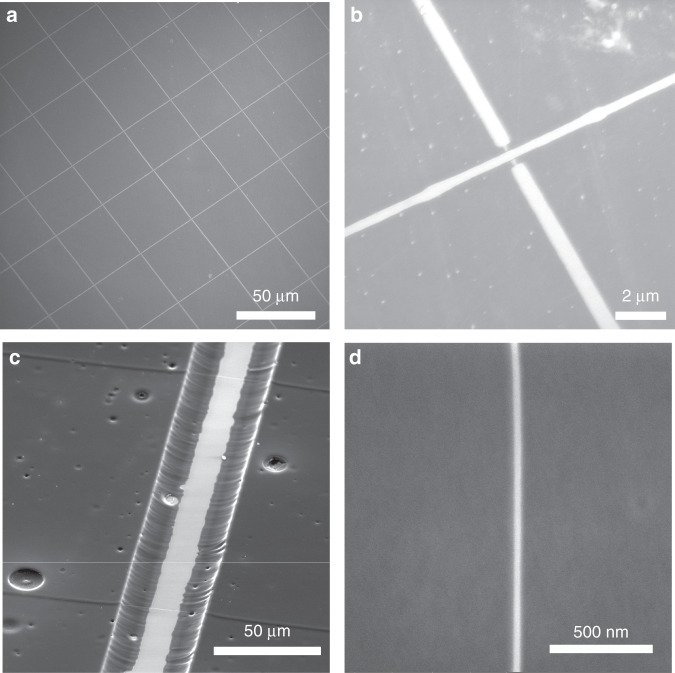
Carbonized polymer wires. **a** Pyrolyzed PAN wire patterns on a flat substrate. **b** Anisotropic shrinkage at the wire intersection. **c** Carbon wire bridges formed on top of a channel structure. **d** Wire of diameter less than 40 nm.

The wires were observed to shrink uniformly during pyrolysis when they were in parallel, but when multiple layers of wires were deposited on top of each other (i.e., crisscross patterns in Fig. 6a), the top wire shrank nonuniformly at the intersections, as shown in Fig. 6b. For a wire in contact with the surface, the shrinkage took place mainly along the height since the bottom of the wire was constrained by the contact that it had with the surface. For a wire suspended between two structures, the wire shrank radially. These differences in shrinkage were the reason for the peculiar shrinkage at the intersection since the wire was in a suspended state on either side immediately adjacent to the first wire. The suspended polymer wires were converted to carbon nanowires by pyrolysis and retained their shape to form bridges across the carbon support structures (Fig. 6c). It is worth noting that the supporting structures used here to suspend the wires were carbonized SU8 in order to avoid breaking the wires due to structural shrinkage during the pyrolysis. Wires smaller than 40 nm were formed due to the combined effect of low-voltage nearfield electrospinning and the shrinkage (Fig. 6d).

The carbon nanowires were characterized with a Renishaw InVia Raman microscope to check the intensities of D (*I*_*D*_), G (*I*_*G*_), and 2D (*I*_2D_) peaks at 1350 cm^−1^, and 1550 cm^−1^, and 2690 cm^−1^, respectively. The ratio of *I*_*D*_ to *I*_*G*_ was observed to be 1 and the ratio of *I*_2D_ to *I*_*G*_ 0.14. These ratios, along with the large width of the 2D peak, indicate the formation of turbostratic carbon, as reported in previous studies^59^.

### Electrodeposition and graphitization

**Fig. 7:**
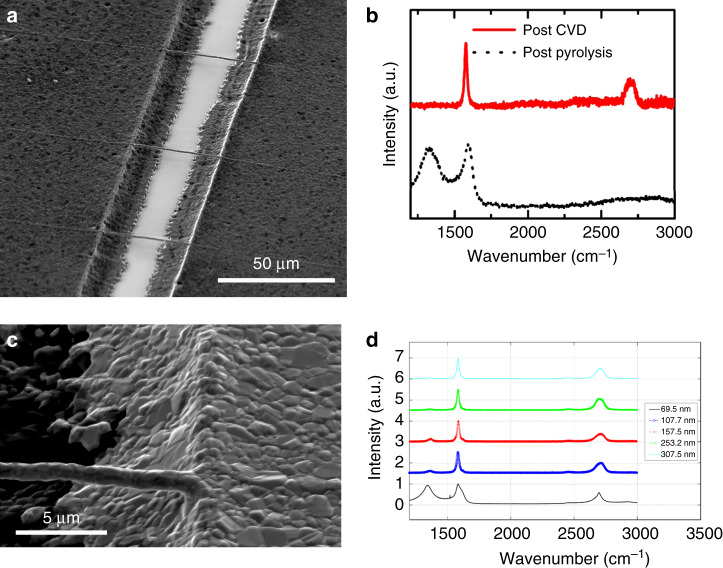
Characterization of graphitized carbon wires. **a** SEM image of suspended Ni-coated pyrolyzed PAN nanowires after CVD at 1000 °C. **b** Raman spectrum of suspended PAN nanowire after pyrolysis (black dotted line) and of suspended Ni-coated pyrolyzed PAN nanowire after CVD at 1000 °C (Red). **c** SEM image showing the microstructure of the electrodeposited nickel (thickness = 307.5 ± 5.9 nm). **d** Raman spectra of graphitic layers formed on nickel layers of different thicknesses. The thicker nickel films showed a higher level of graphitization as indicated by the improved *I*_*D*_(1350 cm^−1^)-to-*I*_*G*_(1550 cm^−1^) ratio.

In order to induce graphitization of the carbon nanowires, a catalyst metal (Ni) was deposited, followed by CVD at 1050 °C. Nickel and copper are the two most widely used catalytic materials for the synthesis of graphene/graphite using CVD. Nickel forms graphite through a precipitation process of ‘soaked-carbon’ due to its high carbon solubility, whereas copper forms graphitic carbon (majorly single-layer graphene) through adsorption process. The carbon solubility of nickel increases with temperature. The high-temperature step is used to “soak” the nickel with enough carbon atoms to form interconnected graphitic layers, which can inherit the shape of the original nickel structure, after cool-down (Fig. 7a). The cool-down step brings simultaneous segregation and precipitation, where the carbon atoms form coherent films^60^. The electrodeposition of nickel was employed here for the goal of developing a low-cost and high-throughput fabrication method for graphitized carbon patterns.

Raman spectra of the nanowires after pyrolysis and after CVD are shown in Fig. 7b. The spectra indicate that CVD at 1000 °C for 2 min was sufficient to induce highly graphitized carbon with a ratio of *I*_2D_ to *I*_*G*_ of ~0.4. Here, the symmetric shape of *I*_2D_ does not indicate the presence of graphite, which is typically identified with a “shoulder”^61^. As the *I*_2D_-to-*I*_*G*_ ratio is low, it is not possible to determine the number of graphene layers^62^, but the Raman spectrum here suggests a material more similar to multilayer graphene than graphite.

The growth of the graphitic film is affected by the microstructure of the nickel film^63^. The surface of the electrodeposited nickel optically appears smooth and mirror-like on the contact pads. Closer examination using SEM reveals roughness attributed by the deposited nickel nanocrystals (Fig. 7c). The heating during the graphitization process results in the modification of the nickel surface to predominantly Ni<111>, the preferred orientation for the graphite growth^64^, which facilitates the graphitization on the deposited film. The thicknesses of the nickel films should also be controlled to produce an appropriate amount of diffused carbon, which leads to the formation of graphitic carbon wires. Experiments were performed to study the electrodeposition process and the effect of the electrodeposited nickel film thickness on the graphitization. The thickness of the deposited nickel was controlled by the duration of the deposition (Fig. S5). Nickel films with a thickness ranging from 70 to 310 nm were made in the current study to investigate the effect of thickness on the subsequent graphitization step. The degree of graphitization of the resulting deposition was evaluated using Raman spectroscopy. It was observed that the samples with thickness more than ~100 nm showed considerably higher degree of graphitization in comparison to the graphite formed on thinner nickel films (Fig. 7d).

## Discussion

A major challenge with prior EMS-based nano writing on flat collectors is the lag in patterning transition when changing patterning directions^65^. With the typical back-and-forth motion on a linear stage to create parallel patterns, the surface placement of the extruded wire lags behind the spinneret as it moves and changes directions. Such spatial and temporal lagging in patterning often causes the deposited wires to overlap and to change diameter, making it difficult to obtain high-resolution writing. The use of a rotating collector overcomes these challenges by avoiding the abrupt directional changes, i.e., back-and-forth motion, and the lagging velocity between the spinneret and the collector (Supporting Document 2). Additionally, compared to a linear stage, the throughput using a rotating collector can be significantly enhanced by enlarging the diameter of the collector, making it viable for large-scale nanomanufacturing. The presented EMS-based nano writing method can attain highly accurate patterned deposition by controlling the rotational and linear speeds.

The throughput of NFES is significantly higher in comparison to those of two-photon lithography and other laser-based nano writing techniques, which offers a maximum throughput of about 5000 μm/s^66^. For instance, the present NFES method allows writing patterns at a resolution (*Δx*) of 8 µm at a throughput of 1 m/s, which is two orders of magnitude higher than other existing nanopatterning approaches. According to the predictive model (Eq. (3)), a highly coveted 1 μm spacing should be achievable with a combination of a low stage speed (~20 μm/s) and a high rotational speed (~1200 RPM) if the aforementioned irregularities are managed. Such high-throughput fabrication of patterned carbon wires is highly desired as there is a lack of a nanomanufacturing system capable of control over micro and macro morphologies for carbon^26^, especially for shapes with dimensions less than 50 nm. Carbon nanowires, both aligned and suspended, have found applications as strain sensors (aligned carbon wires), temperature sensors (suspended wires), and gas sensors (suspended wires)^26^. Apart from these carbon-specific applications, nanowriting of polymer wires also has also been shown to be suitable for the fabrication of nanochannels, enhanced solid electrolyte, light-emitting wires, and nanoresonators ^25,42,67,68^.

Additionally, the patterning method described here may be extended to other polymers to obtain carbon patterns with different properties. The suspended carbon nanowires described here offer a unique advantage for producing graphitic carbon wires with different properties because it is possible to tune the resultant nanowires by controlling the diameter of the template nanowire, the thickness of the electrodeposited metal catalyst, and growth time.

While the presented carbon nanowire fabrication technique has various aforementioned advantages, it has various limits too. This technique limits the fabrication to straight and aligned polymer and carbon wires. Though it is possible to electrospin suspended wires across a large distance, the fabrication of suspended carbon wires is restricted by the geometric parameters. This limitation is due to the transformations that they undergo in the subsequent steps. In the current study, a diameter-to-channel width ratio of ~1000 was obtained. Fabrication of smaller carbon wires is difficult since the polymer wire diameter in the current technique was limited by the low stability of the Taylor cone at low voltages.

## Conclusion

We have developed a high-throughput and scalable NFES technique with a rotating collector, which provides simplified and improved control over the nanowire straightness, diameter, and inter-wire spacing. A simplified model that accounts for various electrospinning parameters was employed to predict the spacing of nanowires and to control their diameter. The polymer wire patterns fabricated with this technique were successfully converted into pyrolytic carbon structures. The new patterning process is capable of producing carbon nanowires of diameter as small as 37.15 ± 1.5 nm and inter-wire spacing of 7.9 ± 2.3 μm with a throughput that outperforms other existing nanopatterning techniques. These dimensions may be further improved by optimizing fabrication parameters based on the above-mentioned predictive models. The proposed technique is also capable of producing suspended wires at a writing speed as high as 1 m/s. The capability to form bridges between structures and the ability to direct them to desired locations using pre-patterned structures further broadens the potential use of this high-throughput wire deposition method. The obtained carbon structures can also be used as a structural template for Ni-catalyzed CVD to obtain highly graphitized structures of the desired shape.

## Materials and methods

**Fig. 8:**
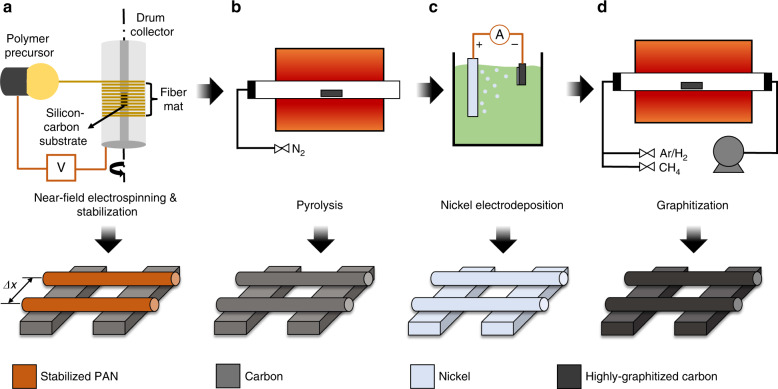
Steps of carbon nanowire fabrication. **a** Electromechanical spinning of polyacrylonitrile (PAN) solution onto a rotating collector to obtain patterned polymer wire deposition. **b** Pyrolysis of stabilized PAN wires inside a furnace to get pyrolytic carbon. **c** Nickel electroplating of the carbon structures to form a catalytic surface for the subsequent chemical vapor deposition (CVD) of graphitic carbon. **d** chemical vapor deposition (CVD) of graphitic carbon.

The electrospinning solution (11% PAN) was prepared by dissolving PAN (150,000 mw, Sigma Aldrich, St. Louis, MO) in dimethylformamide (DMF) by stirring at 50 °C for 24 h. The solution was drawn into a 1 mL syringe with a 30-gauge stainless steel needle. The tip of the needle was fitted with a polytetrafluoroethylene (PTFE) tube to avoid wetting the outside of the stainless-steel tip. The syringe was then attached to a syringe pump. A linear stage with a Prior III controller was used to move the dispensing system at various linear speeds, ranging from 30 to 2400 μm/s. A DC voltage range between 100–1500 V was applied to the tip of the dispensing needle using a high voltage source, which is grounded to the rotating collector (300–2000 RPM) (Fig. 8a). The distance between the spinneret and rotating collector was varied between 0.7 and 10 mm. Either bare silicon substrates or silicon substrates with patterned carbon structures were secured with conductive carbon tape into designated grooves in the rotating drum. A microscope equipped with a camera was used to visually monitor the needle-to-collector distance, the jet initiation, and the stability of the Taylor cone. The wire jet was first initiated by varying the working distance (Supporting Document 1). Aligned nanowires were obtained by moving the syringe pump parallel to the axis of the drum rotation. Experiments were carried out varying the linear speed of the spinneret and the rotational speed of the collector to find the correlation between these two parameters and the inter-wire spacing.

The patterned PAN wires obtained by this technique were pyrolyzed to produce carbon wires. The wires on the silicon substrates were first stabilized at 270 °C for 6 h to ensure high carbon content after pyrolysis. Following the stabilization, a Lindberg Blue M furnace was used for pyrolyzing the nanowires at 1000 °C, under an inert atmosphere, to produce carbon nanowires (Fig. 8b). The resulting carbon nanowires were then compared with PAN nanowires under SEM (Tescan GAIA3) to study the shrinkage caused by pyrolysis. A carbon support structure with a channel width of 40 μm on a silicon dioxide coated Si wafer was used as the collector substrate to fabricate suspended PAN nanowires. This support structure was obtained by pyrolyzing the corresponding SU8 photolithographic pattern. The suspended PAN wires were then pyrolyzed as described earlier. In order to increase the degree of graphitization of the wires, a nickel catalyst was relied on. Ni was uniformly electrodeposited onto the suspended PAN derived carbon wires using an effective current density of 0.3 mA/cm^2^ for 5 min, with a three-electrode setup in an aqueous solution of 1M NiSO_4_ + 0.2M NiCl_2_ + 0.6M H_3_BO_3_ (Fig. 8c). A nickel plate and the suspended carbon wire sample served as the counter and working electrode, respectively, and were maintained at a separation distance of ~2 cm while the Ag/AgCl served as a reference electrode. Clipping the electrode onto the carbon channel allowed the current to conduct through all the connected nanowires in parallel. The nickel-coated nanowires were then gently rinsed in DI water and dried on a hot plate at 70 °C. The nickel-coated nanowires were then loaded into a 1 in Lindberg Blue M quartz tube furnace and evacuated to a pressure of ~10^–2^ torr in a reducing environment of 100 SCCM Ar/H_2_ (5%). The furnace was purged under vacuum/gas for 1 h at ambient temperature and ramped to 1000 °C at a rate of 30 °C/min and held there for 1 h. After 1 h, 0.8 SCCM of CH_4_ was introduced for a growth period of 2 min. The samples were quickly cooled by opening the furnace clamshell and exposing the tube to ambient temperature whilst under reducing environment to obtain graphitized carbon patterns (Fig. 8d).

## Supplementary information

Supplementary Document

Supporting Document 1

Supporting Document 2
