# Supplementary material for: Fabrication of patterned graphitized carbon wires using low voltage near-field electrospinning, pyrolysis, electrodeposition, and chemical vapor deposition
Source: Microsyst Nanoeng. 2020 Jan 13;6:7. doi: 10.1038/s41378-019-0117-7 (PMC8433379; doi:10.1038/s41378-019-0117-7)
Supplement: Supplementary file 1 — Supplementary Document [file 41378_2019_117_MOESM1_ESM.pdf]

Fabrication of Patterned Graphitized Carbon Wires Using Low Voltage Near-field Electrospinning, Pyrolysis, Electrodeposition, and Chemical Vapor Deposition

Supplementary Document

Derosh George[1], Adrian Garcia[4], Quang Pham[5], Mario Ramos Perez[3†], Jufeng Deng[1,2], Michelle Trang Nguyen[4], Tuo Zhou[5], Sergio O. Martinez-Chapa[3], Yoonjin Won[1,5], Chong Liu [2], Roger C. Lo[6*], Regina Ragan[4], and Marc Madou[1]

[1] Mechanical and Aerospace Engineering, University of California, Irvine, USA, 92617

[2] Mechanical Engineering, Dalian University of Technology, China, 116023

[3] School of Engineering and Sciences, Tecnologico de Monterrey, Mexico, 64849

[4] Chemical Engineering and Materials Science, University of California, Irvine, USA, 92617

[5] Materials and Manufacturing Technology, University of California, Irvine, USA, 92617

[6] Chemical Engineering, California State University, Long Beach, USA, 90840

*Corresponding author Roger C. Lo (roger.lo@csulb.edu)

Effect of various electrospinning parameters on the fabrication of suspended wires

Voltage, distance and concentration of the polymer affect the fabrication of suspended wires by using electrospinning. High voltage and short distance results in wires being not suspended as shown in Table S1. On the other hand, suspended wires can be fabricated even at high voltage if the concentration of the solution is sufficiently high as shown in Table S2. Long suspended wires can be formed with 500 V and 11% as shown in Fig. S1.

Table. S1. Array of pictures of wires across a channel depicting the effect of voltage and spinneret-to-collector distance on the ability of the wires to be suspended.

Voltage	Distance = 2.5 mm	Distance = 4.1 mm	Distance = 8.5 mm
1,000V	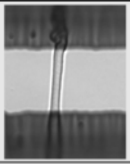 Not suspended	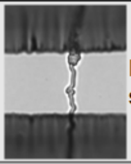 Not suspended	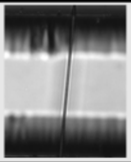 Suspended
900V	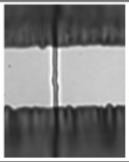 Not suspended	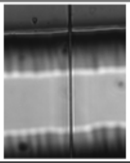 Suspended	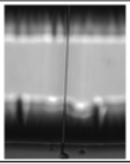 Suspended
800V	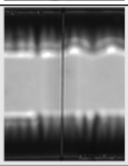 Suspended	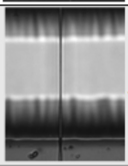 Suspended	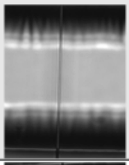 Suspended
700V	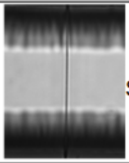 Suspended	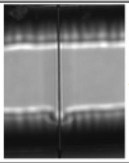 Suspended	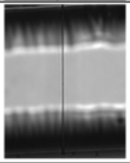 Suspended 20 µm

Table. S2. Array of pictures of wires across a channel depicting the effect of voltage and concentration of PAN solution on the ability of the wires to be suspended.

% PAN	800V	900V	1,000V
7%	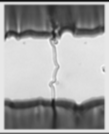 Not suspended	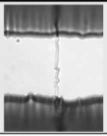 Not suspended	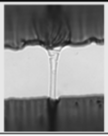 Not Suspended
9%	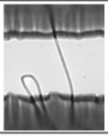 Not suspended	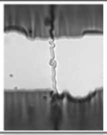 Not Suspended	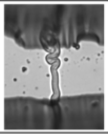 Not Suspended
11%	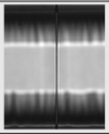 Suspended	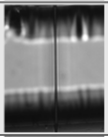 Suspended	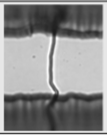 Not Suspended
13%	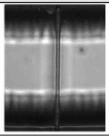 Suspended	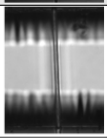 Suspended	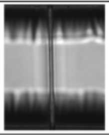 Suspended 20 μm

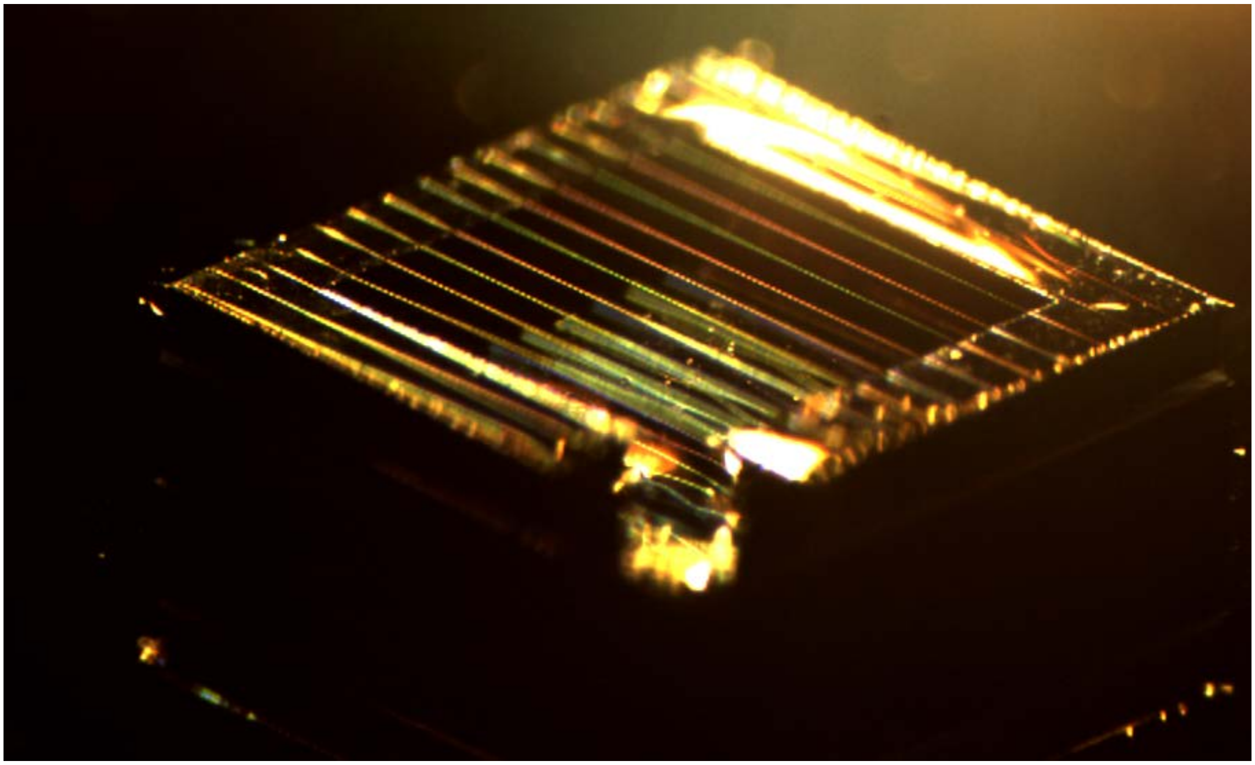

Fig. S1. Wires suspended across a structure of 5mm by 5mm

Effect of electric field on the wire deposition on patterned structures

The effect of the electric field on the deposition was studied by using two different patterns: pillars with circular cross-section and pillars with square cross-section. The simulation carried out using COMSOL Multiphysics showed that the electric field intensities are higher at the edges. This explains the preferential deposition of the incoming wires on the edges of the structures as shown in Fig. S2.

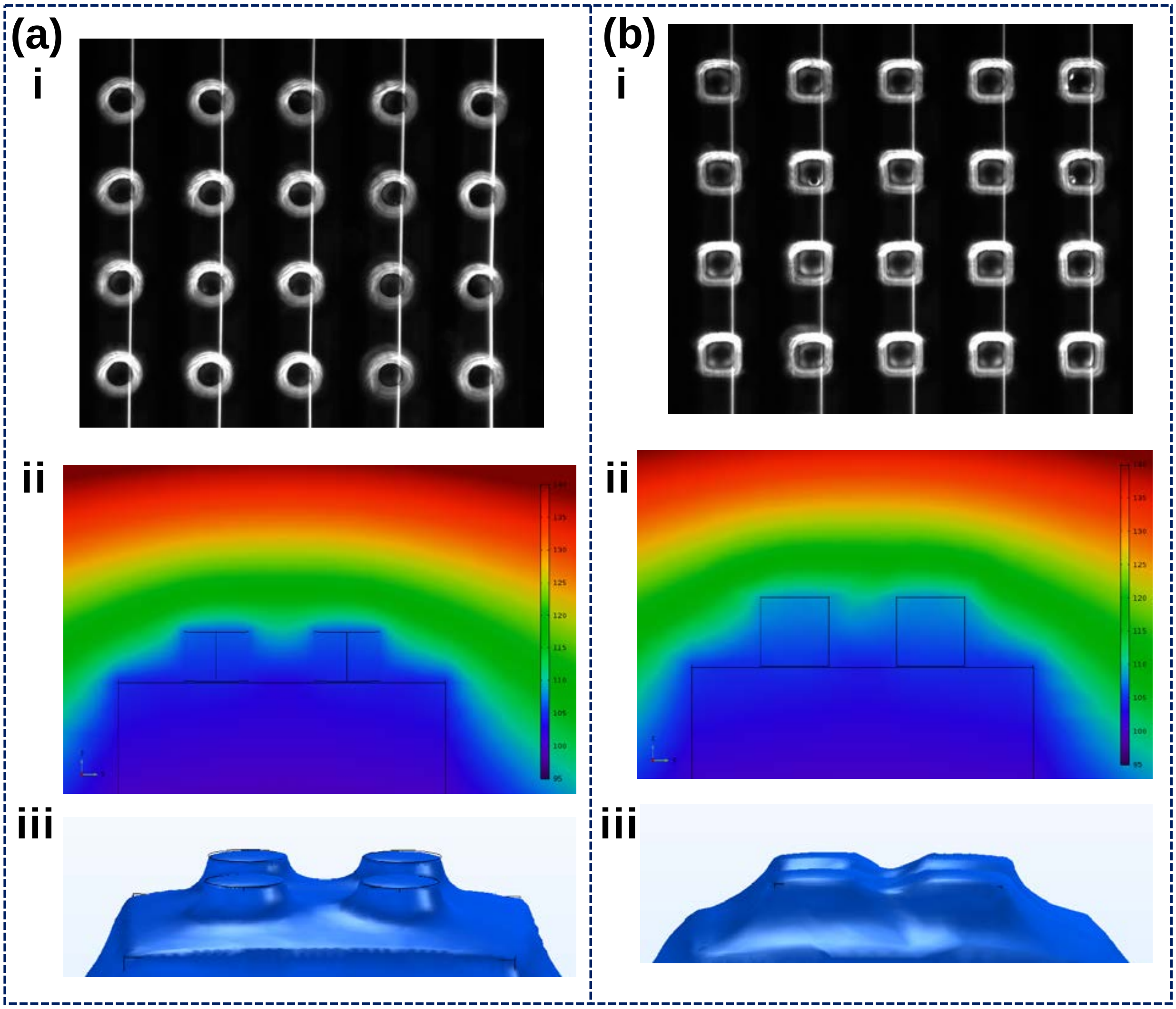

Fig. S2. (a) i. Electrospun PAN wires on pillars with circular cross-section. ii. Electric field intensity on the cross-section of the structures. iii. Isosurface of the electricfield. (b) i. Electrospun PAN wires on pillars with square cross-section. ii. Electric potential at a cross-section of the structures. iii. Iso-surface of the electric potential.

Effect of stabilization and pyrolysis on the suspended wires

Effect of the stabilization and the pyrolysis on the suspended wires are studied by using channels having varying width. It has been observed that the wires that are suspended across wider channels sag and eventually touches the floor of the channel. The data is shown in Table. S3.

Table. S3. Fraction of the total number of the wires in the zone that are suspended.

	Before stabilization	After stabilization	After Pyrolysis
30-40μm	1	0.97	0.87
40-50μm	1	0.43	0.40
50-60μm	1	0.43	0.37

Effect of pyrolysis temperature on the shrinkage

The effect of the pyrolysis temperature on the shrinkage was studied by pyrolyzing the wires at different temperature that are otherwise having same fabrication parameters. The study showed an increasing shrinkage with the pyrolysis temperature as shown in Fig. S3. The shrinkage was observed to be more for the suspended wires in comparison to the wires that are on the surface.

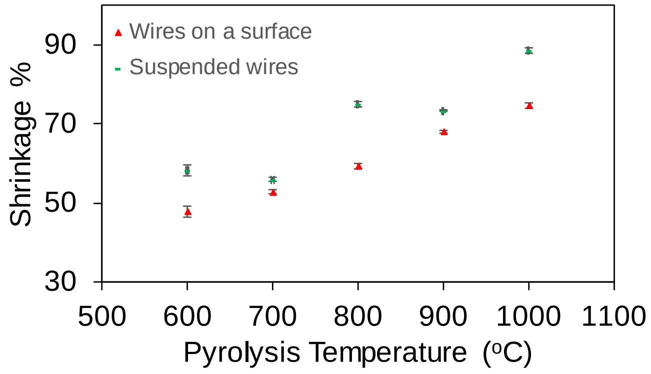

Fig. S3. Variation of the percentage of the shrinkage with the pyrolysis temperature

Controlling the nickel thickness

The thickness of the electrodeposited nickel was controlled by the duration of the deposition. The change in the thickness with respect to the electrodeposition duration is shown in Fig. S4.

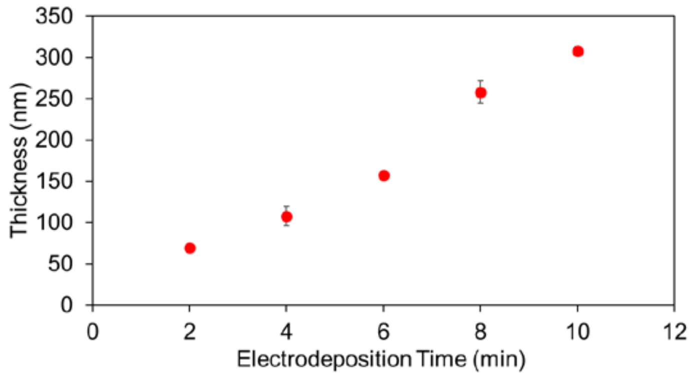

Fig. S4. Nickel thickness variation with electrodeposition time.
