# Supplementary material for: Fabrication of patterned graphitized carbon wires using low voltage near-field electrospinning, pyrolysis, electrodeposition, and chemical vapor deposition
Source: Microsyst Nanoeng. 2020 Jan 13;6:7. doi: 10.1038/s41378-019-0117-7 (PMC8433379; doi:10.1038/s41378-019-0117-7)

Fabrication of Patterned Graphitized Carbon Wires Using Low Voltage Near-field Electrospinning, Pyrolysis, Electrodeposition, and Chemical Vapor Deposition

Supporting Document 2

Derosh George[1], Adrian Garcia[4], Quang Pham[5], Mario Ramos Perez[3†], Jufeng Deng[1,2], Michelle Trang Nguyen[4], Tuo Zhou[5], Sergio O. Martinez-Chapa[3], Yoonjin Won[1,5], Chong Liu [2], Roger C. Lo[6*], Regina Ragan[4], and Marc Madou[1]

[1] Mechanical and Aerospace Engineering, University of California, Irvine, USA, 92617

[2] Mechanical Engineering, Dalian University of Technology, China, 116023

[3] School of Engineering and Sciences, Tecnologico de Monterrey, Mexico, 64849

[4] Chemical Engineering and Materials Science, University of California, Irvine, USA, 92617

[5] Materials and Manufacturing Technology, University of California, Irvine, USA, 92617

[6] Chemical Engineering, California State University, Long Beach, USA, 90840

*Corresponding author Roger C. Lo (roger.lo@csulb.edu)

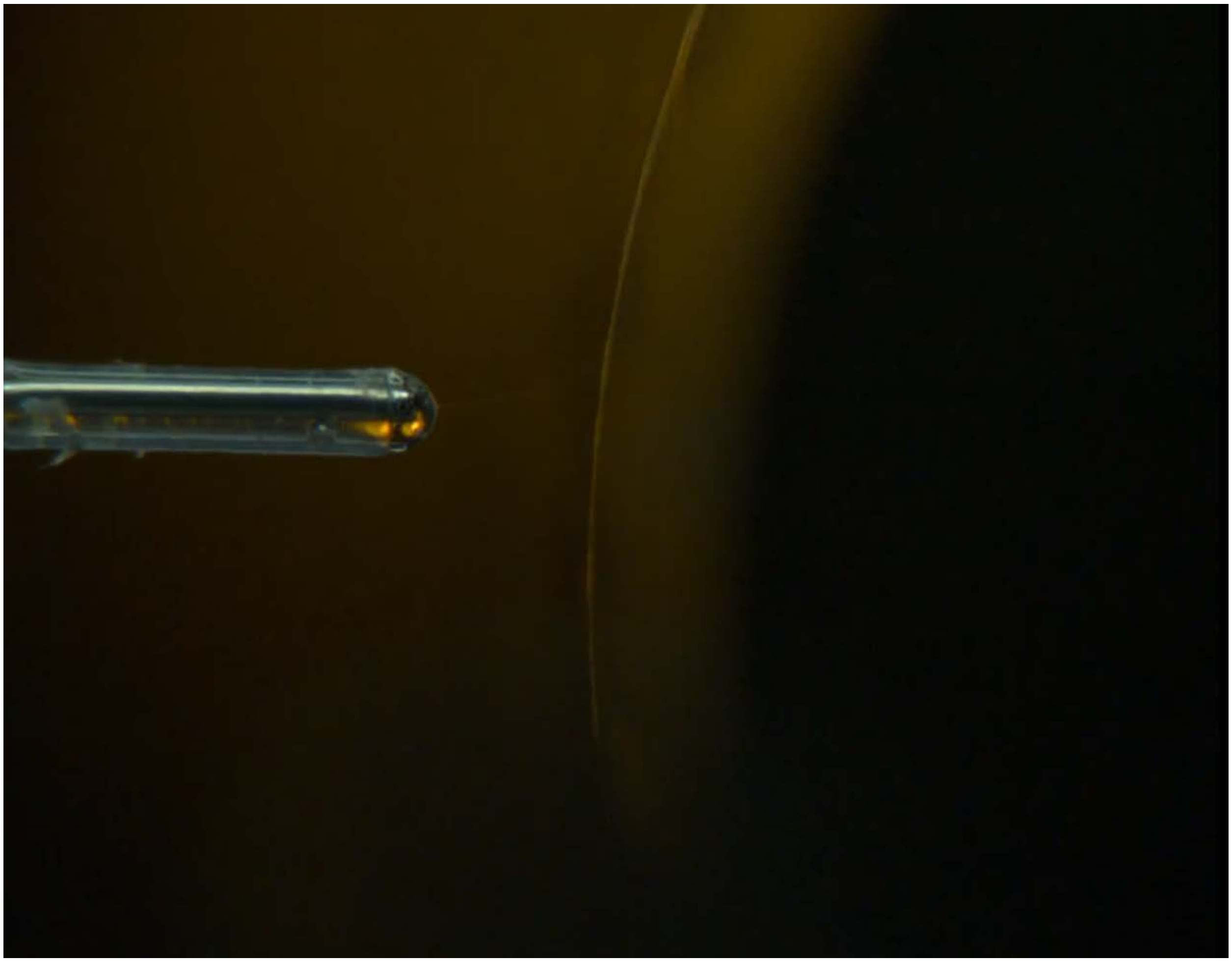

Supplement: Supplementary file 3 — Supporting Document 2 [file 41378_2019_117_MOESM3_ESM.pdf]
